# No evidence of MMR induced trained immunity to prevent SARS COV2: results from a multi-centre RCT

**DOI:** 10.3389/fimmu.2025.1588190

**Published:** 2025-09-16

**Authors:** Sinead Delany-Moretlwe, Hakim-Moulay Dehbi, Izukanji Sikazwe, George Kyei, Kwadwo Koram, Erik Dubberke, Noluthando Mwelase, Dominic Hague, Linda-Gail Bekker, Linda Yun, Annalene Nel, Leon du Toit, Bruce Biccard, Katherine Gill, Chikumbutso Chipeta, Kathryn T. Mngadi, Limakatso Lebina, Reshmi Dassaye, Villeshni Asari, Samantha H. Fry, Edwin Turton, Khatija Ahmed, Kwadwo Kusi, Susan Adu-Amankwah, Roma Chilengi, Joyce Chinyama Chilekwa, Laurence Lovat, Dermot McGuckin, Emilia Caverly, Mary Politi, Ben Swan, Anne DeSchryver, Sherry McKinnon, Ananya Gupta, Gemma Jones, Nicholas Freemantle, Shabaana Khader, Helen Rees, Mihai G. Netea, S. Ramani Moonesinghe, Michael S. Avidan

**Affiliations:** ^1^ Wits RHI, University of Witwatersrand, Johannesburg, South Africa; ^2^ Comprehensive Clinical Trials Unit, University College London, London, United Kingdom; ^3^ Centre for Infectious Diseases Research in Zambia (CIDRZ), Lusaka, Zambia; ^4^ Noguchi Memorial Institute for Medical Research, University of Ghana, Accra, Ghana; ^5^ Washington University School of Medicine, St. Louis, MO, United States; ^6^ Clinical HIV Research Unit, University of Witwatersrand, Helen Joseph Hospital, Johannesburg, South Africa; ^7^ Desmond Tutu Health Foundation, Cape Town, South Africa; ^8^ Medinel, Western Cape, South Africa; ^9^ Department of Anaesthesia and Perioperative Medicine, Groote Schuur Hospital and University of Cape Town, Cape Town, South Africa; ^10^ The Aurum Institute, Johannesburg, South Africa; ^11^ HIV Prevention Research Unit, South African Medical Research Council, Durban, South Africa; ^12^ Family Centre for Research with UBUNTU (FAMCRU), Stellenbosch University, Tygerberg, South Africa; ^13^ Department of Anesthesiology, University of the Free State, Free State, South Africa; ^14^ Setshaba Research Centre, Department of Medical Microbiology, University of Pretoria, Pretoria, South Africa; ^15^ Center for Perioperative Medicine, Department of Targeted Intervention, University College London, London, United Kingdom; ^16^ Department of Internal Medicine, Radboud University Medical Center, Nijmegen, Netherlands

**Keywords:** measles containing vaccines, COVID-19, trained immunity, SARS-CoV-2, prevention, measles, mumps, rubella

## Abstract

**Background:**

Measles-containing vaccines (MCV), by training innate immune cells, are hypothesized to prevent severe acute respiratory syndrome coronavirus 2 (SARS-CoV-2) infection and coronavirus disease 2019 (COVID-19).

**Methods:**

In this international, double-blind, placebo-controlled trial, we randomly assigned adults, 18 years and older, to receive MCV or saline. The primary outcome was polymerase chain reaction (PCR) confirmed symptomatic COVID-19, up to 60 days after intervention. Secondary outcomes were PCR-confirmed symptomatic COVID-19 and serologically confirmed SARS-CoV-2 infection, up to 150 days after intervention.

**Results:**

Of 3411 randomised participants, the modified intention-to-treat population included 1607 in the MCV and 1545 in the saline group. The estimated risk of symptomatic COVID-19 by 60 days was 1.5% in the MCV and 1.2% in the saline group (risk difference, 0.3 percentage points, 95% CI, -0.5 to 1.1; p=0.52). At 150 days, these percentages were 4.1% (65/1585) and 4.1% (64/1544) in the MCV and saline groups, respectively (risk difference, 0.04 percentage points, 95% CI, -1.4 to 1.3; p=0.95). Based on serology results available at 0 and 150 days, 10.6% (100/945) of participants in the MCV and 10.3% (98/951) in the saline group had infection with SARS-CoV-2 over the course of the trial (risk difference, 0.3 percentage points, 95% CI, -2.6 to 3.1; p=0.84). Three patients were hospitalised with COVID-19 disease in the MCV and one in the saline group.

**Conclusions:**

Administering MCVs to stimulate trained immunity did not prevent COVID-19 or SARS-CoV2 infection. Stimulating trained immunity might not be useful for preventing respiratory illness during future pandemics.

**Clinical trial registration:**

https://clinicaltrials.gov/, identifier NCT04333732.

## Introduction

The COVID-19 pandemic, caused by the novel severe acute respiratory syndrome coronavirus-2 (SARS-CoV-2) has led to over 5 million deaths and significant long-term morbidity (in the form of “long Covid” for up to 10% of individuals with prior infection (1). Vaccination to prevent symptomatic infection or reduce symptom impact was an early priority for healthcare scientists. Development of disease-specific vaccines was rapid, but alternative interventions were also considered, to mitigate the risk of disease specific vaccines being ineffective, not immediately available, or not acceptable to some individuals (due to vaccine hesitancy). Therefore, CROWN CORONATION was launched as a platform trial in March 2020 prior to the availability of specific vaccines, with the intention of testing candidate interventions to prevent or mitigate COVID-19.

Accumulating evidence demonstrates that the innate immune system possesses the ability to develop antigen-independent immune memory (2, 3). There is evidence that some existing vaccines (like measles mumps rubella [MMR or MR], oral polio vaccine and Bacille Calmette-Guerin) do not only augment adaptive immunity, but also train innate immune cells to display increased antimicrobial characteristics (4). While vaccine activation of the adaptive immune system leads to classic immune memory specific to the pathogens that are represented in the vaccine, training of the innate immune system theoretically leads to non-specific protection against a broader array of unrelated pathogens (4). There is preliminary evidence from observational studies and systematic review (5–9) that measles-containing vaccines (MCV) could be clinically effective in preventing symptomatic COVID-19.

While several specific vaccines for SARS-CoV-2 have now been developed and have shown substantial protective benefit (10), evaluation of additional preventive measures against covid-19 is still relevant, given that: (i) it takes time to produce and distribute specific vaccines globally; (ii) new strains of the respiratory pathogen repeatedly emerge, and some render specific vaccines less effective (10); (iii) specific vaccines might not confer lasting immunity, since specific antibodies may wane over a period of 4–6 months, as was the case for COVID-19 (10, 11) therefore necessitating booster doses and bringing further logistic and cost challenges; and (iv) non-specific preventions have the potential to provide benefit in future pandemics. MCV are inexpensive, have an established safety record, are stored at 2-8°C, and are readily available across the globe. We report the clinical effectiveness findings from an international, randomised, placebo-controlled phase 3 clinical trial evaluating trained immunity using a single injection of MCV in adults at preventing severe COVID-19 with a 150-day follow-up.

## Materials and methods

### Trial design and setting

CROWN CORONATION was conceptualised as a Bayesian adaptive, pragmatic, participant-level randomised, international multi-centre, placebo-controlled trial; however, after eliminating candidate interventions based on accumulating external evidence (e.g. chloroquine), it transformed into a simple, parallel arm trial that compared MCV against a placebo. A summarised account of the methods is provided here; details are provided in the appended protocol and statistical analysis plan. This manuscript follows the CONSORT guidelines for reporting clinical trials (12).

### Oversight

All participants provided informed consent electronically prior to any study procedures. Ethics committee and other country-specific approvals were obtained at each of the participating sites in the five participating countries (Ghana, South Africa, United Kingdom, United States, and Zambia). The trial was registered prior to enrolment on clinicaltrials.gov (NCT04333732).

### Participants and eligibility criteria

Participants were included if they were adults (18 years or older) at risk for SARS-CoV-2 based on occupational or community exposure and had no clinical evidence of COVID-19 infection, had a mobile phone and access to the Internet for data collection purposes, and were willing and able to provide informed consent via an electronic consent process. Participants were excluded if they weighed outside the range of 50 kg – 120 kg; had a current or previous diagnosis of COVID-19 disease; reported a current respiratory infection; were pregnant; were unable to be followed up for the trial period; had prior receipt of a specific SARS-CoV-2 vaccine; or were planning to receive any other vaccine within 14 days after the study vaccination. Participants were also excluded if they had any contra-indications to receiving MCV namely any confirmed or suspected immunodeficient state, including untreated HIV infection with a CD4 count <200/mL; any malignant disease either untreated or currently undergoing therapy; current use of immune-suppressive drugs; a history of gammaglobulin administration or blood transfusions within the previous 3 months; or allergy to the MCV or its components.

### Randomization

Randomization was stratified by age (<50 and ≥50) and recruitment site. The randomization sequence was computer generated with permuted blocks of size 10. Participants were randomized in a 1:1 ratio to either MCV or placebo. Study staff and participants were blinded to study allocation. The MCV or saline placebo was administered by an unblinded study nurse, who was not involved further in the trial’s conduct.

### Interventions

Participants were recruited using a variety of recruitment strategies. The trial was designed for remote data collection using a web-based electronic case report form (eCRF) system (Sealed Envelope™, London, UK) with the goal of reducing the volume of in-person contacts and the associated risk of SARS-CoV-2 exposure. Once participants registered online, they were sent a link via email to complete an initial participant health questionnaire where they provided basic demographic and eligibility information, including relevant medical history, concurrent use of medication, vaccine history and risk factors for COVID-19. Participants were also asked to provide their own contact information and that of an alternate contact. Permission was also obtained to access other data sources, such as hospital and death records. Following registration, the system sent a notification to the study team who then confirmed that the prospective participant met the eligibility requirements; where needed queries could be addressed by telephone. Following confirmation of eligibility, randomization procedures were triggered through the electronic system and a visit for administration of the investigational medicinal product (IMP) within the next 7 days was scheduled. The participant was considered enrolled once the randomization CRF was completed. An email notification was generated at the point of randomization to inform unblinded pharmacist of the participant identification number and the unblinded allocation. Study staff assisted potential participants to complete online procedures where needed.

At the in-person IMP administration visit, a single dose of MCV or normal saline placebo was administered. During the post-IMP administration observation period (15–30 minutes), participants were counselled about follow up arrangements, and the reporting of COVID-19 symptoms and adverse events. Participants were provided with a self-sample collection kit that included a nasal swab in the event of COVID-19 symptoms and dried blood spot (DBS) cards. Self-collection of mid-turbinate nasal swabs and DBS were demonstrated; a baseline DBS was collected prior to IMP administration. Participants had the option of coming to the site for sample collections. All participants were sent emails with a link to complete monthly health status questionnaires up to 150 days.

Participants were also sent weekly SMS surveys during the first 60 days that asked about possible COVID-19 symptoms and/or adverse events. If a participant reported COVID-19 symptoms, they were prompted to complete an online questionnaire and to undergo testing to confirm the COVID-19 diagnosis, if this was not already done as part of their routine clinical care. Those with a negative test and ongoing symptoms were prompted to undergo a second test. Participants with a confirmed COVID-19 diagnosis were followed up to assess the severity of COVID-19 using a standardised scale and to ensure appropriate linkage to care if needed. Chemiluminescent immunoassay serological testing was done using participants’ baseline and 150 day DBS specimens (13, 14). Infection with SARS CoV-2 during the course of the trial was diagnosed when Ig-G antibodies to the viral nucleocapsid protein were present from the 150 day specimen, but not the baseline specimen (13, 14). If the participants reported any adverse events at the time of vaccination or during the trial, these were graded for severity by study investigators using the Common Terminology Criteria for Adverse Events (CTACAE; v5.0 27-Nov-2017) for grading severity of adult adverse events and assessed for potential relatedness to the IMP by blinded study personnel.

Once specific SARS-CoV-2 specific vaccines were demonstrated to be effective and became available in the country, participants were advised to receive these, regardless of study participation.

### Outcome measures

The primary objective of the trial was to determine the effectiveness of MCV in preventing self-reported symptomatic, PCR-confirmed COVID-19 by day 60 after vaccination. Any of the following were considered a potential COVID-19 symptom: cough, shortness of breath or difficulty breathing, fever, chills, muscle pain, sore throat, new loss of taste or smell, nausea, vomiting, or diarrhoea. Secondary objectives were to determine the effectiveness of MCV in: (i) preventing symptomatic PCR-confirmed COVID-19 by day 150; (ii) mitigating the severity of COVID-19 by day 60; (iii) mitigating the severity of COVID-19 by day 150 (COVID-19 was judged severe if hospital admission or death occurred); and (iv) preventing [symptomatic plus asymptomatic] SARS-CoV-2 infection, based on serologic diagnosis.

### Statistical analysis

A maximum sample size for CROWN CORONATION was determined as follows. We assumed that 30-50% of participants may become infected with SARS-CoV-2 over the course of their participation in the trial. Of these, the percentage of participants experiencing the primary endpoint was uncertain. We used a range of 15-50%. The estimated event proportion was therefore 0.15 * 0.30 = 0.045 (i.e. 4.5%, rounded to 5%) to 0.50*0.50 = 0.25 (i.e. 25%). A 2-arm trial was simulated with an odds ratio for MCV assumed to be 0.7. Three event rates for the control arm were used: 5%, 10% and 15%. We were interested in the lower end of the 5-25% range for the control arm given that a higher sample size is required when the event probability in the control arm decreases for a fixed effect size. The prior distribution of the treatment effect, on the log-odds scale, was a Gaussian distribution such that:

- Prior P(OR < 1) = P(OR > 1) = 50% (i.e. equally likely beneficial as harmful).
- Prior P(OR < 0.5) = P (OR > 2) = 10% (i.e. very large effects in either direction unlikely).

A normal distribution on the log-odds scale, for the intercept prior distribution, was used so that the mean on the natural scale was at 15% and the probability that the rate is greater than 25% was 10%.

The main quantity of interest was P_0_ = Pr(OR < 1 | data). The statistical threshold for P_0_ was 95%, which means that if P0 ≥ 95%, superiority may be concluded efficacy-wise. With 2000–2500 participants per arm, even if the event rate was 5% in the control arm, the study had >80% chance to declare efficacy if the OR=0.7 or less.

We fixed the maximum sample size at 2500 participants per arm, to allow for possible loss-to-follow-up (LTFU), withdrawal, protocol deviation, non-adherence and other methodological challenges.

The primary endpoint was analysed using a Bayesian logistic regression, including as covariates the treatment arm, age (<50 vs. ≥50), and a random effect for site. Prior distributions were the same as in the sample size calculation. In addition, a Cox proportional hazard time-to-event model was used to assess the effect of the intervention on the time to symptomatic laboratory-confirmed COVID-19 infection over the 150-day period since receiving trial interventions. All randomised participant data were included in the Intention-To-Treat (ITT) analysis according to the arm they were randomised to. Subgroup analyses were pre-specified and performed using an interaction term in the model between the treatment arm and pre-specified characteristics of interest, which were: age, HIV status, geographic region, and sex.

## Results

### Trial population

Participants were enrolled into the trial between 9 September 2020 and 15 June 2021. Recruitment was stopped serially at participating sites as access to specific vaccines for SARS-CoV-2 in participating countries expanded, and it was deemed infeasible and unethical to continue enrolment.

**Figure 1:**
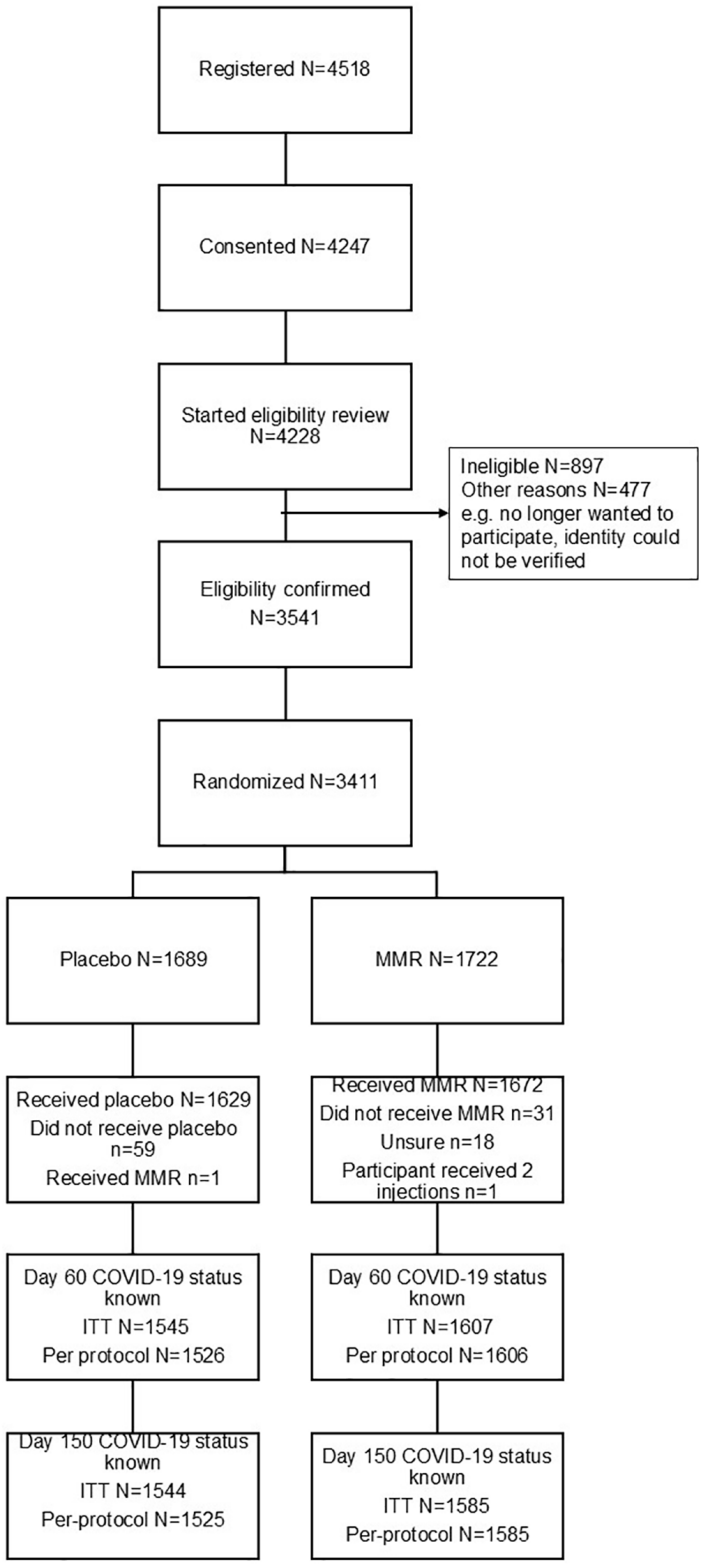
Participant screening, enrolment and follow-up.

Overall, 4518 participants registered on the online system, 4228 were assessed for eligibility and 3541 participants met the eligibility criteria. Of the eligible participants, 3411 participants were subsequently randomised (MCV n=1722, placebo n= 1689) (**Figure 1**). Of these, 3301 received IMP (MCV n=1672, placebo n=1629). One participant in the placebo group received an MCV in error, and we were unable to confirm if placebo or MCV was administered to 18 participants in the MCV group. A total of 3152 (MCV n=1526, placebo n=1607) completed their Day 60 visit and were included in the primary analysis. Follow up questionnaire completion rates were greater than 90% for all visits except day 90 (88.7%).

Participant characteristics are reported in **Table 1**. The majority of participants were recruited at sites in South Africa (73%, 2492/3411), were assigned female at birth (59%, 2025/3411), and were less than 50 years of age (91%, 3105/3411). A very small proportion of participants thought that they had already had covid-19 (4%, 136/3411). Fourteen percent (490/3411) of the trial population were living with HIV.

**Table 1:** Participant characteristics at enrolment, by study group.

Characteristic	Placebo (N=1689) n (%)	MMR (N=1722) n (%)
Country of recruitment		
South Africa	1240 (73.4)	1252 (72.7)
Zambia	284 (16.8)	283 (16.4)
Ghana	75 (4.4)	76 (4.4)
USA	68 (4.0)	85 (4.9)
UK	22 (1.3)	26 (1.5)
Sex assigned at birth		
Female	992 (58.7)	1033 (60.0)
Male	697 (41.3)	689 (40.0)
Age group		
<50 years	1539 (91.1)	1566 (90.9)
50+ years	150 (8.9)	156 (9.1)
Highest level of education		
School (primary/secondary)	1254 (74.2)	1265 (73.5)
Associate degree	73 (4.3)	82 (4.8)
Bachelor degree	118 (7.0)	110 (6.4)
Graduate degree	65 (3.8)	69 (4.0)
Post graduate	102 (6.0)	113 (6.6)
Prefer not to answer	73 (4.3)	80 (4.6)
missing	4 (0.2)	3 (0.2)
Currently a healthcare worker?		
Yes	585 (34.6)	609 (35.4)
No	1104 (65.4)	1113 (64.6)
**Received influenza vaccine in past 12 months**	148 (8.8)	172 (9.9)
Participant believes they had COVID-19, but were not tested		
Yes, probably	19 (1.1)	21 (1.2)
Yes, possibly	47 (2.8)	49 (2.8)
No, unlikely	961 (56.9)	984 (57.1)
No, very unlikely	502 (29.7)	491 (28.5)
Not sure	160 (9.5)	177 (10.3)
Smoking history (cigarettes, cigars, pipes)		
Yes - Current smoker	421 (24.9)	384 (22.3)
No - Never smoked	1194 (70.7)	1237 (71.8)
No - Ex-smoker > 6 months	51 (3.0)	84 (4.9)
No - Ex-smoker <6 months	20 (1.2)	14 (0.8)
missing; n (%)	3 (0.2)	3 (0.2)
Number of days a week with exercise for at least 30 minutes		
None	744 (44.0)	728 (42.3)
one - two	464 (27.5)	495 (28.7)
three - four	251 (14.9)	251 (14.6)
five or more	217 (12.8)	228 (13.2)
prefer not to answer	10 (0.6)	17 (1.0)
missing	3 (0.2)	3 (0.2)
Living with HIV	240 (14.2)	250 (14.5)

### Outcomes

**Figure 2:**
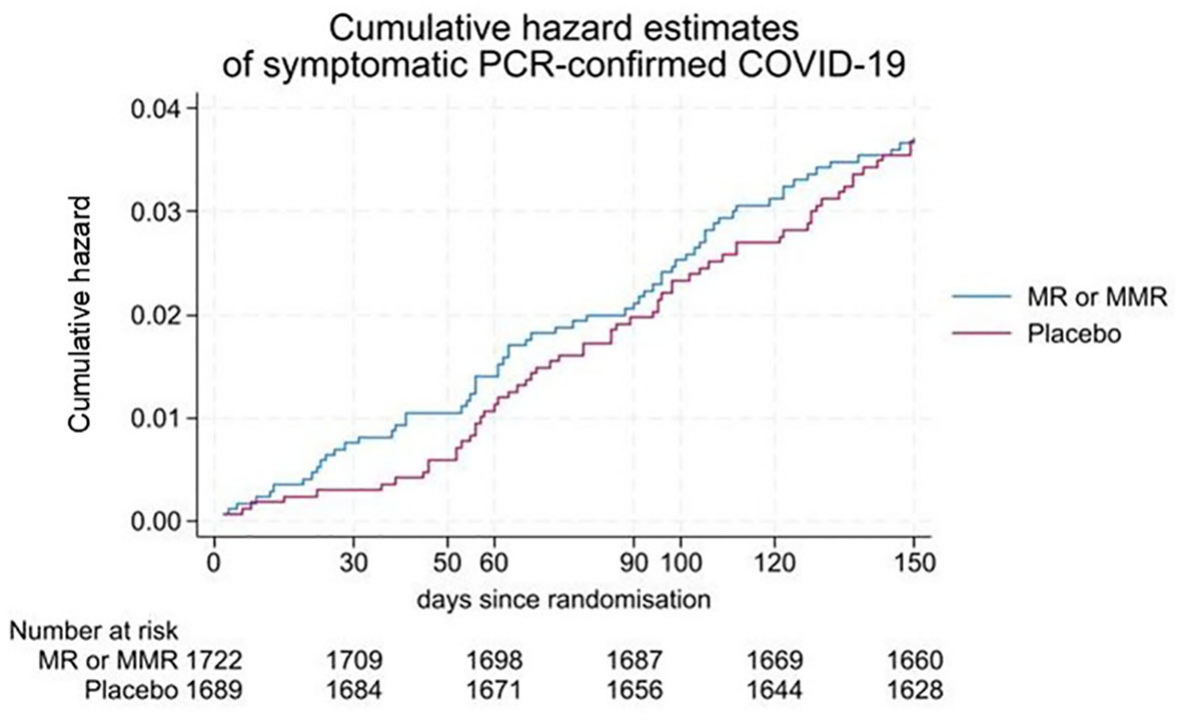
Cumulative hazard estimates of PCR-confirmed COVID-19 over 150 days of follow-up.

**Figure 3:**
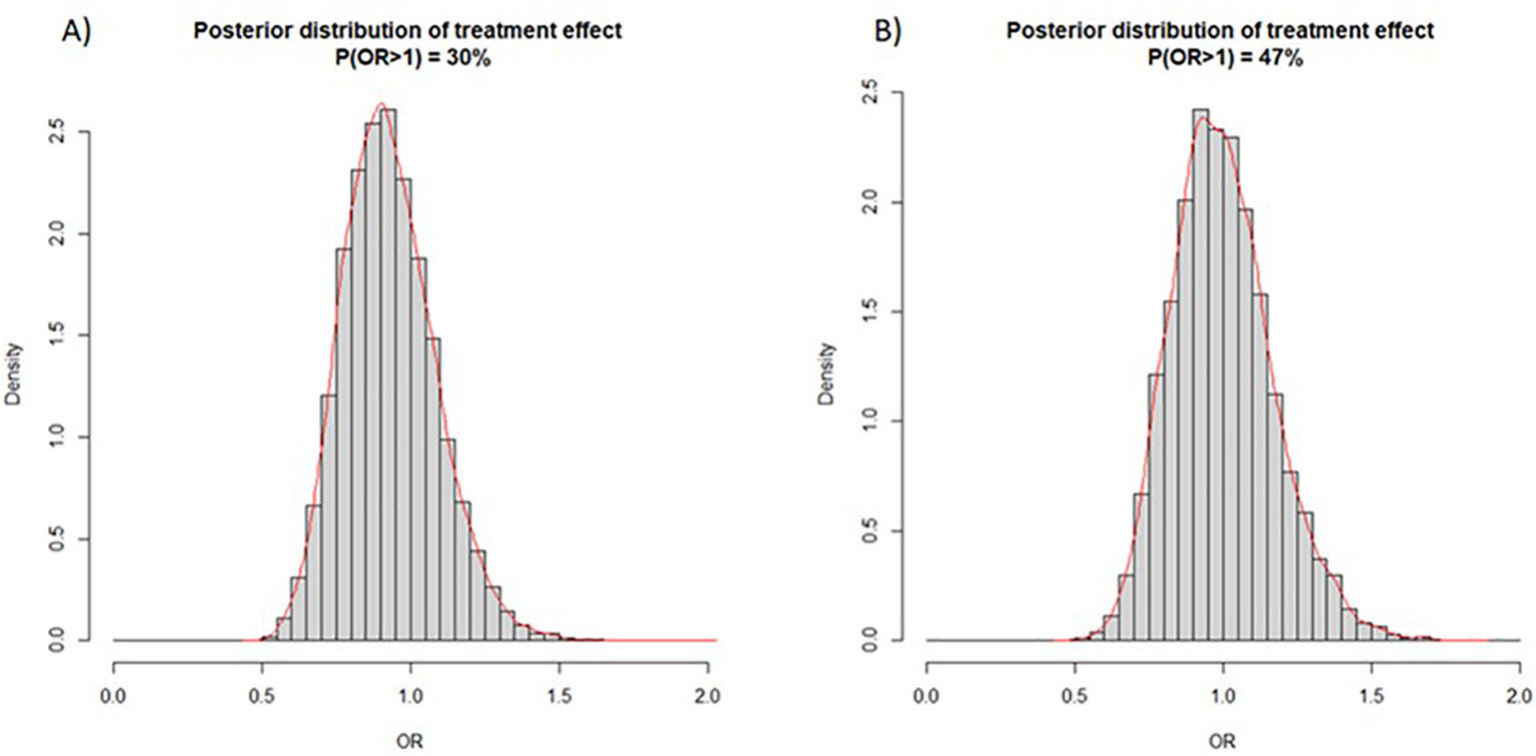
**(A)** posterior distribution of OR according to initial calibration of event probability in the control arm; **(B)** posterior distribution of OR according to revised calibration of event probability centered at 5% (instead of 15%) for the mean of the prior distribution in the placebo arm.

Among those whose COVID-19 status was known at day 60, the proportion with PCR-confirmed symptomatic COVID-19 was 24/1607 (1.5%) in the MCV group and 19/1545 (1.2%) in the placebo group (risk difference, 0.3%, 95% CI, -0.5% to 1.1%, p=0.52). At 150 days, these proportions were 65/1585 (4.1%) and 64/1544 (4.1%) respectively (risk difference, 0.04%, 95% CI, -1.4% to 1.3%, p=0.95). There was no significant decrease over 150 days in the risk of symptomatic PCR-confirmed COVID-19 following measles-containing vaccination (hazard ratio, 1.02, 95% CI 0.71 to 1.44, p=0.93) (**Figure 2**). The posterior distribution of the OR corresponding to the MCV effect is shown in **Figure 3**. Given that the event probability in the control arm was overestimated in the prior distribution compared to the observed data, a second posterior distribution was used where the prior mean for the event probability in the control arm was set at 5% instead of 15% (**Figure 3B**). Using our initial calibration (**Figure 3A**), the posterior probability that the OR is less or equal to 1 is 70%. With the revised prior on the event probability in the control arm, this posterior probability is 53%. Regardless of the prior distribution used, these Bayesian results indicate that there is no evidence of a treatment effect for MCV. Subgroup analyses showed no evidence of heterogeneity of treatment effect in the subcategories of the following variables: age, HIV status, geographic region and sex. Based on serology results available at both 0 and 150 days, 10.6% (100/945) of participants in the MCV group and 10.3% (98/951) of participants in the placebo group had new symptomatic or asymptomatic infection with SARS-CoV-2 over the course of the trial (risk difference, 0.3 percentage points, 95% CI, -2.6 to 3.1; p=0.84). Three patients were hospitalised with COVID-19 disease in the MCV group and one in the placebo group.

### Safety monitoring

Overall serious adverse events (SAE) were rare: there were 10 participants in the MCV group with one SAE, 1 participant in the MCV group with two SAEs, and 5 in the placebo group with one SAE. There were no serious adverse reactions to the intervention.

## Discussion

In this study, we found that stimulating trained immunity using administration of a single dose MCV to adults aged 18 years or older who were at high risk of exposure to SARS-CoV-2 did not reduce the risk of symptomatic, laboratory-confirmed COVID-19 over a follow-up period of 150 days. We also found no evidence for reduction of a combination of symptomatic plus asymptomatic SARS-CoV-2 over the 150-day follow-up period. Despite pre-clinical evidence supporting heterologous benefits of live attenuated vaccines conferred by trained innate immune memory (2–4) and from observational data suggesting lower rates of COVID-19 incidence in those that had received MCV (5–9), we did not observe these effects when evaluated using a rigorous randomised controlled trial design. It is possible that MCV might not be the optimal intervention for training innate immunity. However, results from a multi-centre clinical trial of the BCG vaccine in healthcare workers also did not show a decrease in symptomatic COVID-19 with the intervention (15).

The rapid development of specific COVID-19 vaccines has been one of the greatest academic successes of the pandemic. However, despite the availability of several different specific vaccines, and the international success of making these available to the global population, vaccine hesitancy remains a challenge. The prevalence of hesitancy to COVID-19 specific vaccines ranges from around 1% in the UK to over 20% in South Africa; furthermore, approximately 1 in 8 vaccinated respondents are hesitant to receive booster doses (16). While tackling the causes of vaccine hesitancy remains the key intervention, identifying alternative clinically effective interventions would also be of value and have the potential to reduce mortality and morbidity. Furthermore, if the hypothesis of trained immunity was demonstrated to have scientific validity, and an MCV found to be clinically effective against SARS-Cov2 infection, then this would potentially create a case for prioritising this type of vaccination for evaluation in the event of another respiratory pandemic.

Our findings do not, however, support these notions. Several methodological strengths of the trial support generalizability of its findings, including enrolment of participants in five countries on three continents (the majority were in Africa), almost perfect adherence to group assignment, and excellent retention of participants over the course of the study. Our use of remote monitoring and sample collection was novel and demonstrates the acceptability and feasibility of this type of approach to improve trial participation in more resource- limited settings.

A lower than anticipated incidence of symptomatic COVID-19 raises the potential for a Type 2 error in our results; however the finding of *no-benefit* was consistent over the follow-up period of 150 days, and the lower bound of the 95% CI is only consistent with at most a 30% decrease in the risk of symptomatic COVID-19 with the intervention. The clinical and public health impact of even this hypothetical maximum effect size is unlikely to be important, particularly when compared with the impressive protection afforded by specific SARS-CoV-2 vaccines (10, 17). It is possible that MCV might prevent severe COVID-19 with hospitalisation, but this could not be evaluated, since only four participants were hospitalised during the trial.

In conclusion, based on existing preliminary evidence and multiple unregulated internet-based sources of information, some individuals might have chosen to receive MCV to prevent COVID-19 if it had been found to be clinically effective, particularly given vaccine hesitancy in many communities and expressed safety concerns about specific SARS-CoV-2 vaccines. In the CROWN CORONATION trial, we did not find evidence that MCV trained immunity to prevent symptomatic COVID-19 or infection (whether symptomatic or asymptomatic) with SARS-CoV-2, and, based on these results, administration of MCV to adults is not indicated for these purposes. These data suggest that using live-attenuated vaccines to train immunity to prevent respiratory pathogens of pandemic potential does not seem a feasible strategy for future pandemic responses.

## Data Availability

The original contributions presented in the study are included in the article/supplementary material. Further inquiries can be directed to the corresponding author.
